# Editorial: Metabolic pathways and consequences of malnutrition in children and adolescents

**DOI:** 10.3389/fnut.2023.1282445

**Published:** 2023-09-12

**Authors:** Edyta Łuszczki, Paweł Jagielski, Alejandro Martínez-Rodríguez

**Affiliations:** ^1^Department of Dietetics, Institute of Health Sciences, Medical College of Rzeszów University, Rzeszów, Poland; ^2^Department of Nutrition and Drug Research, Institute of Public Health, Faculty of Health Sciences, Jagiellonian University Medical College, Krakow, Poland; ^3^Department of Analytical Chemistry, Nutrition and Food Science, University of Alicante, Alicante Institute for Health and Biomedical Research (ISABIAL), Alicante, Spain

**Keywords:** children and adolescents, undernutrition, overweight and obesity, diabetes mellitus, nutrition

The science of nutrition was born in the early 20th century with the identification and combination of many known essential vitamins and minerals and their use in the prevention and treatment of diseases associated with nutritional deficiencies, including scurvy, beri-beri, pelagia, rickets, xerophthalmia, and anemia (1). There is now a double burden of malnutrition, characterized by the co-existence of malnutrition from childhood along with overweight and obesity or diet-related non-communicable diseases. Malnutrition and overnutrition are huge challenges for the whole world. Nutritional changes affect every aspect of the endocrine system, leading to serious disorders. Over the past few decades, nutrition has begun to meet the challenge of improving health, including in the pediatric population (2).

Significant global progress has been made in reducing malnutrition in children of all ages over the past 30 years. There is little global data in this age group; recent research reveals new approaches and measures to the current situation. The total number of underweight children aged 5 to 19 years peaked around 2000 and has since been decreasing (3).

On the contrary, obesity has increased in all countries around the world in the past 40 years. From 1975 to 2016, the worldwide prevalence of obesity in children aged five to 19 years increased 8-fold, from five to 50 million girls and from six to 74 million boys (4). Persistent malnutrition and the increase in overnutrition and its consequences in later life have led to a simultaneous burden of malnutrition and overnutrition.

Proper nutrition plays an important role in the lives of children and young people. First, it influences health by reducing the risk of many diseases, including cardiovascular and digestive disorders. On the other hand, malnutrition has far-reaching consequences of both a medical and psychological nature, especially in children. Long-term malnutrition can cause impaired intellectual, emotional, and social development. Malnourished children are less physically fit and have a weak immune system (5).

This special Research Topic includes 4 articles covering the aspects mentioned above.

In the first article, *Perceived quality of care for the management of severe acute malnutrition among caregivers of children under 5 years of age with severe acute malnutrition in Addis Ababa, Ethiopia, 2022: A mixed-method study* authors focused on public health facilities that provide the treatment of inpatient severe acute malnutrition (SAM) in Addis Ababa, Ethiopia. The lack of support and care at higher levels of management in public health facilities and the lack of supplements, separate units, and laboratories were among the factors that prevented the provision of quality care for the treatment of SAM (Adema et al.).

In the study conducted by Domin and Mazur *Nutritional status of a group of polish children with FASD: In a retrospective study*, the authors analyzed data on growth, weight, and nutritional status of children with fetal alcohol spectrum disorders (FASD), which could cause developmental and psychosocial disorders in children. In the FASD group, children (<3 percentile) accounted for 42.31%. Continuous assessment of nutritional status, height, and weight is necessary when caring for children with FASD. This group of patients is often affected by low birth weight, low height, and weight deficiency, which require differential diagnosis and adequate dietary and therapeutic treatment (Domin and Mazur).

The objective of the next article was to explore the association between the prevalence rates of circadian syndrome (CircS) and testosterone deficiency (TD). There is a positive association between the prevalence of CircS and TD in men in the United States. The association becomes more obvious as a result of moderate or vigorous activities (Xiao et al.).

In the last article, *Maternal protein deficiency alters primary cilia length in renal tubular and impairs kidney development in fetal rat* authors showed, with a rat model of maternal protein restriction (MPR), that critical factors of ciliogenesis and critical ciliogenesis factors and β-catenin pathway in the fetal kidneys of FGR and analyzed the impact of aberrant primary cilia on renal tubular epithelium. In conclusion, the authors showed that intrauterine protein malnutrition led to dysregulation of ciliagenesis factors and cilia elongation in the renal tubular epithelium. It could be at least partly responsible for abnormal kidney development and increased risk of hypertension in adulthood (Wang et al.).

In conclusion, the results of the above-mentioned research studies represent an enormous amount of new relevant data on the metabolic pathways and consequences of malnutrition in children and adolescents. Despite all the extant literature and evidence associated with this very relevant topic, the articles published in this Research Topic have clearly shown that there are still many aspects that need to be clarified and understood in relation to the problem of malnutrition in children and adolescents.

## Author contributions

EŁ: Conceptualization, Supervision, Writing—original draft, Writing—review and editing. PJ: Conceptualization, Writing—original draft, Writing—review and editing. AM-R: Conceptualization, Writing—original draft, Writing—review and editing.
